# Comparison of two views of maximum entropy in biodiversity: Frank (2011) and Pueyo et al. (2007)

**DOI:** 10.1002/ece3.231

**Published:** 2012-05

**Authors:** Salvador Pueyo

**Affiliations:** Institut Català de Ciències del Clima (IC3)C/Doctor Trueta 203, 08005 Barcelona, Catalonia, Spain

**Keywords:** Bayesian statistics, biodiversity, idiosyncratic theory, invariant groups, macroecology, maximum entropy, noninformative prior distribution, scale invariance, species abundance distribution

## Abstract

An increasing number of authors agree in that the maximum entropy principle (MaxEnt) is essential for the understanding of macroecological patterns. However, there are subtle but crucial differences among the approaches by several of these authors. This poses a major obstacle for anyone interested in applying the methodology of MaxEnt in this context. In a recent publication, Frank (2011) gives some arguments why his own approach would represent an improvement as compared to the earlier paper by Pueyo et al. (2007) and also to the views by Edwin T. Jaynes, who first formulated MaxEnt in the context of statistical physics. Here I show that his criticisms are flawed and that there are fundamental reasons to prefer the original approach.

## Introduction

The species abundance distribution (SAD) is the frequency distribution of the abundances of the species in a community. In other words, the SAD expresses how many species are rare and how many are abundant. Ecologists have often used simple mathematical expressions to describe the SADs in nature (review in McGill et al. 2007). Many have sought simple mechanisms to explain these simple distributions. However, Pueyo et al. (2007) showed that such simple patterns can also be the result of extremely complex dynamics.

Biodiversity is a result of biological evolution. Natural selection is a powerful mechanism to explore complex fitness landscapes, which comprise many more potential genotypes than the number of particles in the Universe (Wright 1932). The landscapes themselves are continuously modified by the action of coevolution (Kauffman and Johnsen 1991) and environmental changes (Wright 1932). The abundance of a species is largely a result of this complex process, to the extent that niche size and population fluctuations depend on the biology and interactions of the species. Therefore, it is unlikely that a simple, fit-for-all mechanistic model can explain the frequency distribution of the abundances of all the species in a community. Because irreducible (incompressible) complexity represents randomness (see, e.g., Downey and Hirschfeldt 2010), it is plausible that the set of abundances of different species is largely a random set, within few other limits than those imposed by the laws of physics. The irreducible complexity due to the specificities of each species was named “idiosyncrasy” by Pueyo et al. (2007), and is the basis of the idiosyncratic theory of biodiversity.

Once aware of the reasons why the abundances of species could well be “random” to a large extent, we have to express this hypothesis in mathematical terms. To this end, Pueyo et al. (2007) borrowed a tool from statistical physics known as maximum entropy formalism (MaxEnt) and due to Jaynes (1957, 1968, 1978). In informal terms, MaxEnt is a method to find the statistical distribution that is “as random as possible” under some given constraints. One of the most evident constraints is that the total number of individuals cannot be infinite in a finite world. A simple way to introduce this constraint is by setting a limit to the mean number of individuals per species. The result found by Pueyo et al. (2007) from this single constraint was the log-series distribution, which is one of the main classical SADs that ecologist find useful to describe empirical data, since first introduced by Fisher et al. (1943).

Following the notation in Frank (2011), let us use the symbol *y* for abundance and *p* for probability density, in a continuous approximation. (Pueyo et al. 2007 used discrete abundances.) The log-series reads:

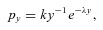
1
where *k* and λ are constants. Small deviations from the log-series are to be expected for several reasons (see section “Deviations from MaxEnt predictions” below). Pueyo et al. (2007) showed that, by taking such small deviations into account, all classical SADs are derived straightforward. In particular, at the limit of very small deviations we obtained a gamma distribution,

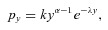
2
which generalizes equation (1) because the additional parameter α can be slightly different from zero.

Surprisingly, at the same time and after the publication of the paper by Pueyo et al. (2007), several other papers appeared that also apply MaxEnt and also obtain the log-series or some very similar distribution (Banavar and Maritan 2007; Dewar and Porté 2008; Harte et al. 2008; Bowler and Kelly 2010; Frank 2011). These papers might seem redundant (except for some results other than the SAD that are obtained in some of them), but they are not, because there are subtle but important differences in their ways to apply MaxEnt. These differences are important for two reasons. First, because the primary aim of these works is to find an explanation for the observed patterns, and the explanation will be wrong if MaxEnt is applied incorrectly, even if the same final result is claimed in all cases. Second, because, if the same methodologies are ever used without previous knowledge of the results to be expected, their predictions are unlikely to be correct unless the methodologies are correct.

Such subtle differences among the methods in different papers are a jigsaw for anyone attempting to apply MaxEnt in an ecological context. Further advance will be difficult unless they are carefully compared. The present paper is a contribution to this task. In fact, the approaches by Banavar and Maritan (2007) and by Bowler and Kelly (2010) are very similar to the approach in Pueyo (2006, Appendix B), which was already discussed in Pueyo et al. (2007, Appendix A). Among the rest of papers, I gave priority to Frank (2011) because he included an appendix with a critique of Pueyo et al. (2007). He went beyond and also stated that his own way to apply MaxEnt is better than that of Edwin T. Jaynes, who first developed this method in the context of statistical physics. The aim of the present paper is to reply Frank's criticisms by showing that there are fundamental reasons to prefer the original approach rather than his version of MaxEnt, and also that he does not give an accurate description of the contents of our paper.

## The Maximum Entropy Formalism

As already mentioned, the maximum entropy formalism (MaxEnt) can be described as a method to find a distribution of probability {*p_y_*} that is “as random as possible” within some explicit constraints. This distribution maximizes (using, again, Frank's notation)

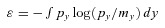
3
subject to the relevant constraints (see details in Pueyo et al. 2007). In this equation, {*m_y_*} is also a distribution of probability, known as “non-informative prior distribution.” A crucial but often overlooked feature of MaxEnt is that it cannot be applied without previously determining this distribution, which represents full randomness (free of constraints) and depends on the type of variable. The constraints always include the trivial one that the sum of probabilities is 1, which I take for granted hereafter.

For example, take the case in which *y* represents a coordinate in space (instead of species abundance as in the rest of the paper). There is broad agreement in that a “random” position corresponds to a uniform distribution in all of the axes of coordinates, that is, *m_y_* is uniform in this case. If we maximize ? in equation (3) with no extra constraint we obtain *p_y_*=*m_y_*. It is well known that molecules reach their maximum entropy when they are uniformly distributed in space. Consider, however, the case of the atmosphere, where the molecules are subject to the Earth's gravitation field. Jaynes (1978) maximized equation (3) under the constraint that the sum of the potential energies of all the molecules has a given value, and obtained the well known “barometric formula,” according to which the density is maximum at the bottom of the atmosphere and decreases exponentially with height. This is a good approximation to reality, although some other, site-specific constraints are needed to reach an even more realistic distribution.

To predict a frequency distribution using MaxEnt we thus need (1) to determine the correct prior distribution and (2) to know the constraints to which the system is subject. We can add a third point, which is the treatment of the deviations from the result obtained by applying MaxEnt. In each of these three aspects there are notable differences between Frank (2011) and Pueyo et al. (2007), summarized in Table 1. They will be discussed sequentially in each of the following sections.

**Table 1:** Differences between two ways to apply MaxEnt to predict the species abundance distribution.

	Pueyo et al. (2007)	Frank (2011)
Noninformative prior distribution	Determined by the invariant groups criterion	Assumed uniform
Ecological constraint	Mean of the abundance *y*	Mean of *y*+*b*log(*y*), where *b* is fitted a posteriori
Deviations from maximum entropy	Taylor series expansion	Not considered

## The Prior Distribution

The first step to apply MaxEnt is to find the prior distribution *m_y_* (eq. 3) expressing absence of information. In the example above, I assume that the prior distribution for the coordinates in space is uniform. There is a widespread belief that the uniform also expresses absence of information in all other contexts. Frank's (2011) paper is an instance (and so is Harte et al. 2008; Harte 2011), as he assumes a uniform prior distribution *m_y_*∝ 1, because, he claims, the uniform is “the most random pattern with the highest entropy, and the pattern that lacks any information.” However, there is consensus in the statistical literature in that this is not the case, even though there is no such consensus in the most correct alternative (Kass and Wasserman 1996).

The problem is most evident for continuous variables, because every continuous variable *y* is equivalent to an infinite number of other variables, for example, *x*= log(*y*), *z*=*e^y^*, *v*=√*y*. If *y* contains no information, none of these three other variables contains any information either, but at most, only one of them can be uniform, so we find a contradiction.

In the context of MaxEnt, this problem was already mentioned in the first paper about this method (Jaynes 1957), but only a partial solution was given at that point. A complete solution was presented in Jaynes (1968). Neither Frank (2011) nor Frank and Smith (2010) give any reason why the problem and the solution found by Jaynes should be ignored, only stating without proof that Jaynes’ method “does not always give the correct answer” (in Frank and Smith, 2010, p. 301).

The solution found by Jaynes (1968) is named “invariant groups” method and is based on the symmetries of each problem (see also Jaynes 1973; Pueyo 2011). For example, when we are seeking a noninformative prior for SADs in general, we are not specifying whether these SADs correspond to a whole continent or to one's backyard. If *m_y_* were different in each of these cases, the prior distribution would include information that was not specified in the enunciate of the problem. Therefore, it would not be a noninformative distribution. The noninformative prior distribution of SADs has to be scale invariant, that is,

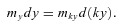



The solution of this equation is 

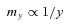
4
(see also Shipley 2009). This distribution is named log-uniform because, among all the possible parameterizations of *y*, the one with a uniform distribution is *x*= log(*y*). As noted, we have been assuming a continuous set, but, in fact, species abundances constitute an infinite discrete set. Pueyo et al. (2007) sought the result of applying the same symmetry to discrete abundances *n*, and also found a prior proportional to 1/*n*. Besides scale invariance, we could think of other symmetries implied in a situation of absence of information about the SAD (e.g., location invariance), but I am aware of none contradicting this result.

Pueyo et al. (2007) abided by the criterion of invariance to derive the noninformative prior distribution of the SAD unambiguously, from a noninformative spatial arrangement of individual organisms. Therefore, there is no justification for Frank's (2011) claim that our derivation of the prior was based on “particular ecological assumptions.” Furthermore, we showed that strong deviations away from the noninformative spatial arrangement did not alter the result. Of course these deviations involved relatively specific ecological assumptions, but were not needed for the main result. Even if they had been needed, this approach would have been more reliable than the unjustified choice of a uniform *m_y_*.

In a more recent paper, Frank and Smith (2011) accept that the uniform distribution is not conserved under a change of variable. They state that the prior should be uniform at the “scale” at which the information “dissipates.” However, this choice does imply assumptions about particular mechanisms. Furthermore, these authors give no reason why the information should dissipate on a linear “scale” in the case of species abundance.

**Figure 1:**
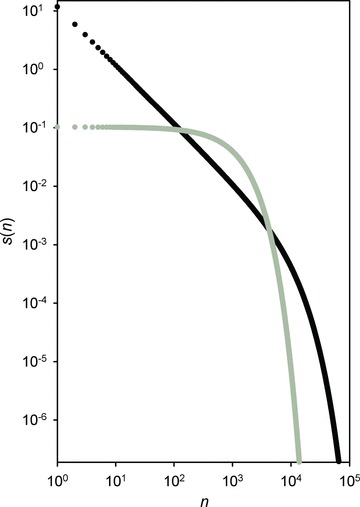
Illustration of the importance of using the correct noninformative prior distribution when applying the maximum entropy formalism (MaxEnt). The two curves are predicted species abundance distributions (SADs) obtained by applying MaxEnt, with the only assumptions that there are 107 species and no more than 112,352 individuals, as is the case for a dataset of Mediterranean diatoms analyzed by Pueyo (2006). The expected number of species with *n* individuals is *s*(*n*). The black plot assumes that the noninformative prior is log-uniform, and the gray one assumes that it is uniform. According to the logical criteria in Pueyo et al. (2007), the correct prior is log-uniform. Pueyo (2006) shows that the resulting SAD agrees with the empirical observations. If, instead, the logical arguments proved that the correct prior is uniform, the difference between the unrealistic gray plot and the realistic black plot would indicate that we are ignoring some important constraint.

In this context, a correct choice of prior is extremely important. Figure 1 compares the results of using a uniform or a log-uniform prior when the only constraint is the mean abundance. The predicted frequency distributions are completely different: exponential in the first case and log-series in the second. For example, the expected number of singletons (species with one single individual) differs by two orders of magnitude in this instance.

Among the two plots in Figure 1, only the one that results from the prior used by Pueyo et al. (2007) is realistic (Pueyo 2006; see also McGill et al. 2007). However, this observation, in itself, says little about which prior is correct. The determination of the noninformative prior can never be based on empirical data: it is based on logics (discussed in Pueyo 2011). In this section, I have summarized the logics behind the assertion that the prior can only be log-uniform (eq. 4) in this context (more details in Pueyo et al. 2007) and I have shown that there is no logical basis for the uniform prior, which Frank suggests as an alternative to our choice. If the opposite were true, the large difference between the gray curve in Figure 1 (which follows from the uniform prior) and empirical SADs would not affect the determination of the prior but would mean that we have ignored some important constraint. Frank (2011) introduces a constraint ad hoc to obtain a realistic result, as treated in the next section.

## Constraints

In spite of the diverse features of the species in a community, they do not cover the whole space of possibilities that could be imaginable a priori, either because some of these possibilities are incompatible with the laws of physics, because they are systematically disfavored by natural selection, or because of historical reasons. From the idiosyncratic theory, we expect SADs to display maximum entropy, but subject to some minimum constraints.

As mentioned, a constraint on the mean species abundance (which has to be finite for physical reasons) is enough to give quite a realistic SAD. This is true when the prior has been obtained by applying the standard method of invariant groups, but not when assuming a uniform prior as Frank (2011) does, as is evident from Figure 1. In order to fill the gap from the nonrealistic SAD shown in gray in Figure 1 to the realistic SAD shown in black in the same figure, Frank (2011) constrains the mean value of the function:

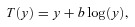
5
where *b* is a parameter that is fitted a posteriori. He calls this constraint “log-linear scale.”

The constraint on *T* belongs to a class of constraints put forward by Frank and Smith (2010), which they name “constraints on measurement scale.” In their approach, the diversity of these constraints is intended to replace the diversity of the prior distributions used by Jaynes (1968). However, while Jaynes’ choice of prior distribution follows a strict logic (the invariant groups method), which warrants that it will not be affected by the desired result, Frank and Smith's choice of constraint is ad hoc. More importantly, these constraints do not solve the problem for which Jaynes’ method was designed. Whether or not these constraints are used, the assumption that a uniform *m* is universally valid leads to contradictions. Among the different parameterizations of *y* mentioned in the previous section, a uniform *m* combined with a “log-linear scale” gives equivalent results when applied to *x*, *y*, and *v*, but not when applied to *z*, as is the case for an infinite number of other parameterizations. As discussed in the previous section, this limitation was recognized in a later paper by Frank and Smith (2011) but no satisfactory solution was given.

The “log-linear scale” constraint chosen by Frank (2011) in the case of SADs is a clear instance of the ad hoc character of the choice of “measurement scale” constraints in Frank and Smith's (2010) method. Frank (2011) recognizes that he ignores the reason why this constraint should hold. As an example of possible cause, he puts forward a specific population dynamics model, in spite of his stated goal of using “recent advances in maximum entropy to strengthen the argument that many different mechanistic hypotheses lead to the same common SAD pattern,” and accusing Pueyo et al. (2007) of relying on “particular ecological assumptions,” which was not the case.

## Deviations from MaxEnt Predictions

Biological species are not completely idiosyncratic, and, besides mean abundance, there might be many other constraints, if less important and more difficult to anticipate. Furthermore, communities will often display fluctuations away from their state of reference (analogous to fluctuations away from thermodynamic equilibrium in isolated physical systems). Pueyo et al. (2007) used a standard technique known as Taylor series expansion to perturb the maximum entropy distribution in equation (1) (details in Pueyo 2006). This treatment uncovered a direct link between the log-series and the other classical SADs. The minimum perturbation leads to a gamma distribution (eq. 2), which generalizes equation (1) because the additional parameter α can differ from zero. Pueyo et al. (2007) also mentioned that equation (2) is equivalent to the maximum entropy distribution when, in addition to the arithmetic mean of *y*, the geometric mean is constrained.

Frank (2011) states that, “to get the gamma from the log series,”Pueyo et al. (2007)“realize that the geometric mean has to be allowed to vary independently of the arithmetic mean, so, ad hoc, they allow the geometric mean to vary independently.” This is not a faithful description of the content of our paper. Finding a gamma was not our goal and we did not reach it by introducing constraints ad hoc. It was the result of introducing small perturbations in the log series by using Taylor series. We mentioned that this was equivalent to constraining the geometric mean, but this was not its justification. We stated (Pueyo et al. 2007, p. 1022): “The equations of MaxEnt allow us to concisely describe the terms in the Taylor series as constraints on the distribution. Nevertheless, as we have not established these constraints a priori, our ultimate reason to expect these modifications is the Taylor series and not MaxEnt.”

Frank (2011) coincide with Pueyo et al. (2007) in that the gamma distribution gives a good description of SADs, but instead of attributing this distribution to small deviations from the state of maximum entropy, he claims to have obtained it by maximizing entropy. Since his “log-linear scale” constraint (eq. 5) combines the arithmetic mean and the geometric mean, it gives rise to a gamma distribution (eq. 2) with arbitrary α. This constraint was chosen ad hoc, so his criticism of our paper in fact applies to his own choices. Furthermore, his approach does not explain the reason why, in empirical observations, deviations from α= 0 (in eq. 2) are typically small. In contrast, this is the natural expectation if empirical SADs result from small perturbations of the log-series (where α= 0) as suggested by Pueyo (2006) and Pueyo et al. (2007).

## Final Remarks

In a recent paper, Frank and Smith (2011) propose a general classification of probability distributions. Acting as a reviewer of the present paper, Frank's main claim is that my comments about his SADs’ paper (Frank 2011) are not valid unless I propose another classification of distributions. However, I do not think that this is necessary or even appropriate.

In logical terms, the contribution by Frank and Smith can be classified into three parts: the basic premises (the general approach, first introduced in Frank and Smith 2010), some conclusions for ecology (the reinterpretation of the SADs, in Frank 2011), and some conclusions for general statistics (the classification of probability distributions, in Frank and Smith 2011). The object of the present paper is to discuss the basic premises and the ecological conclusions. The fact that some other conclusions have also been drawn from the same premises tells nothing about whether or not these premises are correct. (Note that Frank and Smith 2011 is a deductive paper, with no empirical test involved.) Therefore, my proposing a classification of probability distributions would shed no light on the problem treated in this paper. Furthermore, this would belong to a journal of statistics rather than ecology and evolution. An aspect in which Frank and Smith (2011) depart from the premises in Frank and Smith (2010) has been discussed in the section “The Prior Distribution” above.

In addition, it should be clear that the first object of the present paper is to reply Frank's criticisms of Pueyo et al. (2007) and of Edwin T. Jaynes, the father of the maximum entropy formalism. Frank states that this paper is not valid unless I address his “full published work,” but Frank and Smith never addressed the whole contribution by Edwin T. Jaynes and, as mentioned, did not even give any argument for their claim that Jaynes’ method “does not always give the correct answer.”

In this paper I have shown that Frank's (2011) description of Pueyo et al.'s (2007) paper was not accurate, and, more importantly, that there are strong reasons to prefer the approach by Jaynes and by Pueyo et al. over Frank's attempt to replace it. In coming papers I will extend the comparison to other published versions of MaxEnt in community ecology.
